# Sibling Death after Being Thrown from Window by Brother with Autism: Defenestration, an Emerging High-Risk Behavior

**DOI:** 10.1155/2015/463694

**Published:** 2015-08-03

**Authors:** Osman Sabuncuoglu, Mustafa Yasin Irmak, Nagehan Ucok Demir, Duygu Murat, Can Tumba, Yuksel Yilmaz

**Affiliations:** ^1^Department of Child and Adolescent Psychiatry, School of Medicine, Marmara University Hospital, 34899 Istanbul, Turkey; ^2^Marmara University School of Medicine Foundation, Academic Hospital, 34662 Istanbul, Turkey

## Abstract

Children diagnosed with autistic spectrum disorders (ASD) may have serious behavioral problems such as aggression, self-injury, and violence. However, the literature on ASD either overrules any correlation between aggression and ASD or maintains the fact that the efforts to link them have so far been inconclusive. Although severe forms of violence are extremely rare in children with autism, there are a few cases reported in the literature with significant harm to siblings. We hereby report an 8-year-old boy with ASD who caused the death of his sibling by throwing her out of the window. Shared similarities of all defenestration cases indicate a pattern of high-risk behavior threatening the survival of minors. We recommend precautions against this high-risk behavior in children with ASD.

## 1. Introduction

Autistic spectrum disorders (ASD) refer to a group of pervasive developmental disorders that are persistent from childhood to adolescence and characterised by impairments in social communication, social interaction, and restricted range of interests [1]. Compared to normal children, children with autism may have serious behavior problems such as aggression, self-injury, and violence [2–4]. Consequences of violence in physical and sexual assaults may comprise most severe forms. However, there is limited information about prevalence of violence associated with ASD.

In the course of the psychiatric disorders, varying degrees of violence occur. Many mental health problems including anxiety, depression, posttraumatic stress disorder, eating disorders, and psychosis are associated with violence. Some recent reviews [5] and studies of hospitalised individuals [6] suggest that it is psychiatric comorbidity with ASD such as psychosis or substance misuse that increases an individual's vulnerability towards interpersonal violence. The authors of the earliest known review of the link between Asperger Disorder (AD) and violent crime concluded that no such connection exists [7]. In a more recent review, the determination was that the link is inconclusive and is supported by only 11 of 147 studies on AD and violence when the strictest inclusion criteria are used [8].

Violence in ASD is associated with deficits in theory of mind abilities, abnormal repetitive interest, high sensory reactivity, naïve interpersonal communication, and reactive violence to negative environmental stimuli. Individuals with ASD have significant difficulties reading social cues and can harm others due to negative environmental stimuli and sensory hypersensitivity. Despite reports of homicide by adults with ASD existence, there is only one report of homicide committed by a child with ASD. Mukaddes and Topcu [2] reported a 10-year-old girl with a diagnosis of autistic disorder, who caused the death of her 6-month-old sister by throwing her out of window in Turkey. Her aggressive-impulsive behavior had a persistent pattern. She had a history of epilepsy and was frequently exposed to physical abuse. Furthermore, in her book published in Turkish, Mukaddes notes 2 additional cases with ASD who committed similar sibling-throwing behaviors one of which also ended up with death [9].

Such cases should be reported because of fatality and the possibility of permanent harm to others. In this paper, we would like to report an 8-year-old boy who had a history of causing the death of his 18-month-old sister by throwing her out of window.

Before authoring this report, we obtained written and oral consent from the father of the child provided that their identity and certain details would not be disclosed.

## 2. Case Report

An 8-year-old boy with autistic disorder was admitted because of aggression, violence, and poor behavioral control. Prior to the admission, he was seen by the author who is child neurologist and, after rigorous neuroradiologic and metabolic work-up, no abnormal neurological condition was detected.

The family was living in a remote metropolitan city in Anatolia. In order to seek a second opinion about the diagnosis, the child was admitted during a visit to Istanbul. The parents, both of them in their midthirties, were first cousins. There was an older 11-year-old sister. The history of the patient revealed uneventful pregnancy and ordinary vaginal birth. The child's early development was delayed and, consequently, a diagnosis of autistic disorder had been given. The child was attending a special school for autistic children and a center for special education for 4 years at the time of admission. Previous trial of risperidone 0.75 mg daily was discontinued as a result of severe sedation. The child was reported to be on a trial of Cortexin [10] which is not officially licensed in Turkey.

In psychiatric assessment, lack of speech and communication was observed. The father was constantly trying to control the child's sudden anger outbursts and he had facial bruises left by the child's punching. Mental disability along with autistic features presented that the child had no preconception of consequences of his behavior. On the Modified Turkish Version of Autism Behavior Checklist (ABC) [11], the child's score was 42, indicating a clinical condition of low-functioning autism.

The most tragic part of the case history was the death of 18-month-old young sister after being thrown out of the window by the patient, about 11 months before the latest admission. The injured child spent an overnight in the intensive care unit and eventually died as a result of intracranial hemorrhage. It was reported that the child with autism was not under the influence of any medication at the time of the incident.

Afterwards, a criminal investigation was initiated. The child was identified as the main suspect yet the investigation was dismissed on the grounds of incompetence due to insanity and being below the age of criminal responsibility. Consequently, the file was closed.

After a comprehensive psychiatric assessment, the child's diagnosis was confirmed as autistic spectrum disorder with comorbid Mental Retardation and, besides attending special education, further recommendations were given to the parents in coping with the challenging behaviors of the child. Follow-up care was to be carried out by local doctors as the family was living in a remote location.

## 3. Discussion

In youngsters with ASD, deficits in theory of mind abilities, abnormal repetitive interest, high sensory reactivity, and ineffective interpersonal communication may lead to violent behaviors and, as in the present case, even loss of life may be the consequence. It must be noted that death of a child by their sibling is an extreme form of sorrow for the family members and the community. As far as we know, the present case appears to be an important addition to the preadolescent children with ASD causing the death of their siblings. Similarities shared by all cases may indicate the potential of a dangerous pattern of behavior against minors by children with severe autism.

It has been shown that falls are the most common type of childhood injury presenting to emergency departments and falls from a height represent an important subgroup with serious consequences [12, 13]. A correct and focused description of the incident in the present case refers to the term “defenestration,” which means the act of throwing someone or something out of a window [14], in order to remove an adversity. Defenestration carries a very high risk of early mortality related to uncontrollable brain injuries and severe associated lesions in children under the age of 6 [15].

It appears that there is no room for controversy regarding the legal handling of the present case. The child with autism is clearly below the age of criminal responsibility. Prosecution of individuals with autism requires specific attention to core legal concepts like competence to stand trial, capacity to commit a criminal offense, sentencing issues, and evidentiary issues [16]. It is expected that the more severe the impairment is, the more likely an accused with autism will be able to successfully raise an autism-related defense [16].

Since there is no evidence of adverse childhood experiences (e.g., child abuse) in this case, it appears that defenestration behavior stems from the neurodevelopmental disorder which unfolds after birth. The most distinctive symptoms that led to the death of the sibling seem to be a high level of aggression, low level of impulse control, and severe form of disability. Throwing away objects is a common and sometimes excessive behavior in children with autism [17]. It is very likely that the patient acted on his impulses to remove an unwanted object in his sight without anticipating its consequences. Absence of theory of mind may disable the patient from anticipating the grieve consequences when an infant is defenestrated. Certain aspects of infant behavior, like crying and screaming, may trigger aggressive reactions in individuals with ASD because of sensory hypersensitivity.

Because of severe behavioral difficulties and lack of speech, the present case falls into the low-functioning end of the autistic spectrum disorders. Although this appears to be in contrast to some previous reports of violence associated with high-functioning autism and/or Asperger Disorder, we should bear in mind that the characteristics of the present case (e.g., age and defenestration) significantly differ from those of earlier reports [8].

The present case shares several similarities with the cases reported by Mukaddes and Topcu [2] and later by Mukaddes [9]. All children had the same diagnosis of ASD and, in all cases, aggression is targeted towards siblings who were vulnerable minors. The act that caused the death of all siblings was the same, that is, defenestration, when there was no adult around. In the present case, several intrafamilial adversities were not evident, unlike the case reported by Mukaddes and Topcu [2]. Thus, we will argue that similarities shared by all cases indicate the potential presence of a very dangerous behavior in children with ASD, particularly in cases with poor impulse control and severe disability. As a final note, all cases residing in Turkey are another dimension that merits attention, yet we are not aware of any possible cultural factor relating to this.

Pharmacologic treatment is strongly recommended to ensure behavioral control over violent children with autism. Individuals with ASD who have poor impulse control should never be left alone with minors who are unable to defend themselves. Constant supervision must be a rule. Window guards, which significantly reduce falls, should be secured [18]. On the other hand, it is a well-established fact that many individuals with ASD are victims of violence and caution is required in order not to label them as potential murderers. Despite the limitation that we lack follow-up data of the present case, we believe that the pattern of a potential dangerous behavior must be reported and highlighted in order to improve professional care. The precautions in this paper should be viewed as steps that aim to create safer environments for all children and families.
